# Can Adverse Childhood Experiences Heighten Risk for Problematic Internet and Smartphone Use? Findings from a College Sample

**DOI:** 10.3390/ijerph18115978

**Published:** 2021-06-02

**Authors:** Myriam Forster, Christopher J. Rogers, Steven Sussman, Jonathan Watts, Tahsin Rahman, Sheila Yu, Stephanie M. Benjamin

**Affiliations:** 1 Department of Health Sciences, College of Health and Human Development, California State University, Northridge, Los Angeles, CA 91330, USA; jonathan.watts.126@my.csun.edu (J.W.); stephanie.benjamin@csun.edu (S.M.B.); 2 Institute for Health Promotion and Disease Prevention Research/Keck School of Medicine, University of Southern California, Los Angeles, CA 90032, USA; rogerscj@usc.edu (C.J.R.); ssussma@usc.edu (S.S.); tahsin.rahman.683@my.csun.edu (T.R.); sheilayu@usc.edu (S.Y.)

**Keywords:** adverse childhood experiences, Internet addiction, problematic smartphone use, social support, college students

## Abstract

Background: College students are among the heaviest users of smartphones and the Internet, and there is growing concern regarding problematic Internet (PIU) and smartphone use (PSU). A subset of adverse childhood experiences, household dysfunction [(HHD) e.g.; parental substance use, mental illness, incarceration, suicide, intimate partner violence, separation/divorce, homelessness], are robust predictors of behavioral disorders; however, few studies have investigated the link between HHD and PIU and PSU and potential protective factors, such as social support, among students. Methods: Data are from a diverse California student sample (*N* = 1027). The Smartphone Addiction Scale—Short Version and Internet Addiction Test assessed dimensions of addiction. Regression models tested associations between students’ level of HHD (No HHD, 1–3 HHD, ≥4 HHD) and PSU and PIU, and the role of extrafamilial social support in these relationships, adjusting for age, gender, ethnicity, SES, employment loss due to COVID-19, and depression. Results: Compared to students reporting no HHD, students with ≥4 HHD had twice the odds (AOR: 2.03, 95% CI: 1.21–3.40) of meeting criteria for PSU, while students with 1–3 HHD and ≥4 HHD had three and six times the odds of moderate to severe PIU (AORs: 2.03–2.46, CI:1.21–3.96) after adjusting for covariates. Extrafamilial social support was inversely associated with PIU and moderated the HHD–PSU association for students with 1–3 HHD. Conclusion: Students exposed to HHD may be especially vulnerable to developing behavioral addictions such as PSU and PIU. Extrafamilial social support offset the negative effects of HHD for PSU among the moderate risk group; implications for prevention efforts are discussed.

## 1. Introduction

College students, and young adults generally, are the most likely population to go online and own digital devices such as smart phones and are among the heaviest users of the Internet [1,2,3]. Historically, students’ Internet activities have centered around research, communicating, browsing, and shopping [4]; however, with the proliferation of online applications and social media platforms, there has been a dramatic increase in the amount of time students spend online. While there are many advantages to accessing the Internet, there is a growing interest in the relationship between digital behaviors and young adults’ psychological wellbeing, the potential for excessive Internet use, and whether the features of compulsive use are consistent with attributes of other addictions [5,6,7]. Excessive or compulsive Internet use has been associated with loneliness and isolation [8,9,10], online gaming, gambling, and sex addictions [11,12], and prospectively with risk behaviors such as substance use [13]. Given the increasing concern over the potential for, and effects of, excessive use, investigating factors that exacerbate or inhibit vulnerability for problematic or compulsive Internet use has become an important area of Internet research, and one that has implications for the health and wellbeing of college students.

In a similar vein, devices such as smartphones that increase connectivity to the Internet have become essential tools for most Americans. Smartphones are mobile phones that perform many of the functions of a computer, have touch screen interface, and offer access to applications. Over 90% of American young adults now own a smart phone [3], and many of them acknowledge that they could not imagine their life without one [14]. Although smartphones offer easy access to the Internet and enhanced communication [15,16], there is mounting evidence of a potential for problematic/addictive smartphone use (PSU) [17,18,19]. As scientific understanding of behavioral addictions evolves, addictions such as Internet gambling have been added to the Diagnostic and Statistical Manual of Mental Disorders (DSM-5). Although problematic Internet use (PIU) and PSU are not currently included in the DSM-5, research suggests a strong potential for compulsive use among younger populations, and there is mounting evidence suggesting that compulsive use is linked to psychological disorders [20,21,22], stress [23,24], sleep difficulties [25], and compromised academic performance [7,26,27,28]. Among students internationally [29,30,31,32,33], PIU and PSU prevalence are estimated to be between 10% and 44% and between 5% and 28%, respectively [34,35,36].

Problematic or compulsive Internet and smartphone use has been conceptualized as excessive, maladaptive use that a person is unable to regulate despite negative consequences, such as failing to meet responsibilities and obligations, a preoccupation with the Internet, and concealing and feeling guilty about use—features that are typical of substance dependence and addiction disorders [37,38,39,40]. According to Griffiths’ six-component model of addiction, addictions—whether substance or behavioral—share common characteristics; salience, mood modification, tolerance, withdrawal, conflict, and relapse that develop through similar biopsychosocial pathways [41,42]. Salience indicates a preoccupation with the Internet or digital device and “craving” the behavior; mood modification refers to engaging in the behavior to elevate mood or to avoid thinking about other aspects of life [43]; tolerance suggests that the amount of engagement in the behavior required to produce a desired experience increases over time [44]; withdrawal suggests the presence of physiological and psychological symptoms when decreasing or abstaining from the behavior; and conflict refers to compromised personal relationships, work or educational goals.

Among the most robust predictors of substance and behavioral addictions are adverse childhood experiences (ACE), a set of highly correlated traumatic and negative events experienced prior to age 18 that heighten risk for compromised health in adulthood [45,46]. A subset of ACE, and the focus of this study, is household dysfunction (HHD), defined as parent/caregiver stressors or behaviors (e.g., parental divorce, mental illness, suicide, substance use, intimate partner violence, homelessness, and incarceration). Family-based stressors (ACE) are a form of toxic stress that over time can damage a child’s stress response system, the processes that limit the intensity and duration of the stress response [47,48], and alter neurological structures and functioning [47,48]. In conjunction with these physiological changes, unpredictable and inconsistent parenting and home environments can impair the cognitive and emotional processes involved in self-regulation [49], self-esteem [50], and decision making that in turn increase vulnerability to maladaptive coping behaviors and addiction [51,52,53].

The effects of HHD on processes that promote or inhibit healthy behaviors may be especially relevant during the college years when students must learn to balance the demands of their academic goals, adapt to their increasing autonomy from family, assume greater responsibilities, and begin planning for their future. While the college experience can be a time of exploration and transformation, the enhanced susceptibility for engaging in risky behaviors during this transition period can threaten students’ ability to successfully obtain a degree, develop enduring social bonds, and participate in the work force [54,55,56,57,58].

There is substantial evidence linking family environments to socioeconomic disadvantage, academic challenges, and psychiatric morbidity over the life course [59,60], and studies have investigated the association between specific aspects of the family environment (e.g., sexual abuse or family violence and parent–child communication) and PIU, PSU, or individual Internet behaviors [61,62,63,64,65]. This work has found that young people who report a form of maltreatment or family discord are at greater risk for PIU and PSU or specific Internet behaviors (e.g., online gaming) than young adults without this history [36,66,67,68]. However, despite evidence that cumulative HHD is highly predictive of early adult developmental deficits, compromised mental health, and maladaptive behaviors, especially behaviors with an addictive potential [69,70,71,72], research assessing the association between household stressors (HHD) and health-compromising behaviors among US college students has lagged behind that conducted in other countries [29,30,31,32,33]. Within this limited body of work, there is evidence of a graded relationship (e.g., as the number of adversities/stressors increase, so does the risk for poorer outcomes) between HHD and depression, substance use, and alcohol-related consequences [73,74,75,76].

In contrast, and in the context of resilience, the buffering hypothesis suggests that if individuals facing chronic adversity have sufficient social or individual resources, they will be less vulnerable to maladaptive coping behaviors and experience fewer of the short and long-term pathogenic effects of stressful events [77]. In fact, developing social relationships is an important aspect of adolescent and early adult development [78,79] and perceived social support, a central feature of health and wellbeing across the lifespan [80,81,82], and it can promote wellbeing, reduce morbidity and mortality [83,84], and diminish the harmful effects of ACE [85].

Despite (a) young adults’ vulnerability for risky behavior, (b) the high prevalence of ACE documented in student populations, (c) and the pervasive use of the Internet and digital devices among this age group, the association between cumulative HHD and PIU and PSU has not been well studied in the US college population. Although research suggests that social support and relationships with important others can offset the negative effects of early adversity, whether social support buffers the association between HHD and PIU and PSU has yet to be determined. To address these gaps in the literature, the present study investigated the association between HHD and PIU and PSU with two widely used, validated instruments that have established thresholds of problematic Internet and smartphone use. The Smartphone Addiction Scale—Short Version (SAS-SV) developed by Kwon et al. [17] captures four of the six components of addiction [41]—conflict, withdrawal, tolerance, and salience—while the Internet Addiction Test (IAT) developed by Young [39] assesses salience, withdrawal, secrecy/concealment, lack of control, conflict, mood modification, and developing online relationships to replace relationships that may be missing or unfulfilling in offline settings. We hypothesized that students with a history of HHD, and especially multiple household stressors, would have higher odds of (H1) PIU and (H2) PSU. We also anticipated that perceived social support from friends would be protective such that (H3) students with a history of HHD who report high perceived social support from friends would have lower odds of PIU and PSU than their peers with the same history of HHD, but who have low perceived social support from friends.

## 2. Materials and Methods

A sample of 1027 students enrolled at a public university in Southern California agreed to participate in this study. An email containing a link to the survey was sent to randomly selected undergraduate and graduate classes in October 2020 and was open through December 2020 while the university maintained distance learning due to the COVID-19 pandemic. Approximately 60% of classrooms that were invited agreed to participate. Students who participated received information on study objectives and procedures and consented online. All aspects of the study were approved by the university IRB.

### 2.1. Measures

Sex was assessed with one question, “What sex were you assigned at birth, such as on an original birth certificate?” and coded male = 1 and female = 0. Ethnicity was measured with one question that asked, “How do you usually describe yourself?” and coded Non-Hispanic White = 0, Non-Hispanic Black = 1 Mexican = 2, Other Hispanic = 3, Asian = 4, and other and Bi/Multiracial = 5. Age was a continuous variable, and COVID-19 financial hardship was a continuous variable calculated by subtracting the hours worked after the COVID-19 shutdown from the hours worked prior to the shutdown. Due to the strong correlation between depression and PIU and PSU [6,7,86,87], we included an indicator of depression derived from the 10-item Center for Epidemiological Studies Depression Scale [88] to reduce confounding.

Perceived social support from friends was assessed with items from the Multidimensional Scale of Perceived Social Support developed by Zimet [89] that has been validated in diverse populations. For the purposes of the present study, only perceived social support from friends was used in the present study. Sample items include “My friends really try to help me,” and “I can count on my friends when things go wrong.” Response options were “Agree” coded = 1 or “Disagree” coded = 0. Responses were summed, with higher scores indicating greater perceived social support from friends.

Household dysfunction (HHD) items were adapted from the original ACE study [90]. Questions asked respondents if, prior to age 18, their caregivers misused alcohol, illegal drugs, or prescription drugs, engaged in intimate partner violence, suffered from mental illness, attempted suicide, were separated/divorced, incarcerated, homeless, or if they had stayed in a shelter. Household dysfunction was coded 0 = no HHD, 1 = 1–3 HHD, and 2 = ≥4 HHD.

Problematic smartphone use was measured using the Smartphone Addiction Scale Short Version (SAS-SV) developed by Kwon et al. [17]. SAS-SV assesses four of the six components of a widely used six-component model of addiction [41]: conflict, withdrawal, tolerance, and salience. Respondents selected options on a six-point Likert scale ranging from “Strongly Disagree” to “Strongly Agree” to statements such as “Missing planned work due to smartphone use” and “Constantly checking my smartphone so as not to miss conversations between other people on Facebook or Twitter”. As recommended by the developers, we adopted a score of 32 out of 60 as the threshold to distinguish high-risk (problematic/addictive) users. PSU was coded = 1 nonproblematic use = 0 (Cronbach alpha = 0.88).

Levels of Internet addiction were assessed using the 20-item Internet Addiction Test (IAT) [39]. The IAT assesses characteristics (e.g., salience, excessive use, neglect of work, anticipation, lack of control, neglect of social life) that are consistent with salience, mood alteration, withdrawal, secrecy, and conflict. Sample items include “How often do you neglect household chores to spend more time online?”, “How often do your grades or school work suffer because of the among of time you spend online?”, “How often do you try to cut down the among of time you spend online and fail?”, and “How often do you try to hide how long you’ve been online?” Response options are on a 5-point Likert scale 0 = Not applicable, 1 = Rarely, 2 = Occasionally, 3 = Frequently, 4 = Often, and 5 = Always (Cronbach’s alpha = 0.92). As recommended by developers, scores ranging from 0 to 30 reflect normal levels of Internet usage, scores ranging from 31 to 49 indicate the presence of mild levels of Internet addiction, scores between 50 and 79 indicate the presence of moderate levels of Internet addiction/compulsivity, and scores between 80 and 100 reflect severe levels of Internet addiction. Because only 13 students reported severe Internet addiction, we collapsed the moderate and severe categories, resulting in a three-level variable coded normal use = 0, mild = 1, and moderate to severe = 2 Internet addiction.

### 2.2. Analytic Plan

Missing data, a common problem in survey studies, ranged from 3.0% to 8.0% (Table 1). Multiple imputation by chained equations (MICE), an approach outlined by van Buuren, Boshuizen, and Knook [91], was conducted to account for missing items. MICE does not assume a joint multivariate normal distribution, and unlike single imputation, multiple imputation builds into the model the uncertainty/error associated with the missing data and instead uses a separate conditional distribution for each imputed variable [92,93,94]. We used M = 10 imputations as recommended for valid estimates with <10% missing data that reduce sampling error due to imputations [91,95,96]. The uncertainty parameter estimation in the missing data case is the average of the parameter estimate obtained over M = 10 imputed datasets. The variance of estimation is partitioned into the within-imputation variance, which captures typical sampling variability, and the between-imputation variance, which captures the estimation variability due to missing data. Missing data are described in Table 1.

A multivariable logistic regression model assessed the associations between HHD and PSU adjusting for age, sex, race/ethnicity, financial hardship due to COVID-19, depression, and peer social support. A second model that included an interaction term (HHD∗peer social support) assessed whether perceptions of peer social support could offset the negative effects of HHD for PSU. Results are reported as odds ratios (OR) and 95% confidence intervals (95% CI).

An ordinal logistic regression model assessed the association between HHD and mild, and moderate to severe IA adjusting for age, sex, race/ethnicity, financial hardship due to COVID-19, depression, and social support from friends. A second model included an interaction term (HHD*peer social support) and tested whether peer support buffered the association between HHD and mild and moderate to severe levels of IA. To understand the association between HHD and each level of IA (normal use, mild addiction, and moderate to severe addiction), the delta method was used to calculate the probability of normal use, mild IA, and moderate to severe IA for HHD categories (none, 1-3 HHD, and ≥4 HHD). All analyses were conducted using STATA v. 15 [97].

## 3. Results

Over 88% of study participants were between 18 and 29 years old, and approximately 12% of students were 30 years old or older. The majority of respondents were female (78%), and approximately one third of students identified as Mexican (38%), followed by Bi- or Multiracial (17%), Non-Hispanic White (16%), Asian (12%), other Hispanic (12%), and Non-Hispanic Black (4%). Students from the seven colleges on the campus participated. The percentages of respondents enrolled in the College of Education; Science and Mathematics; Engineering and Computer Science; and the Social and Behavioral Sciences were consistent with the university’s published data while the College of Arts, Media, and Communication; Business Economics; Humanities; and Health and Human Development were over or under-represented by 9–15%. Slightly over half the sample acknowledged experiencing some form of household dysfunction, 47% reported one to three household stressors, and 9% reported over four. Regarding Internet and smartphone use, over half of the sample could be classified as having normal levels of Internet use, about 34% as mild IA, and 10% were in the moderate to severe PIU category. Approximately 25% of students were at high risk for PSU (Table 2).

**Figure 1:**
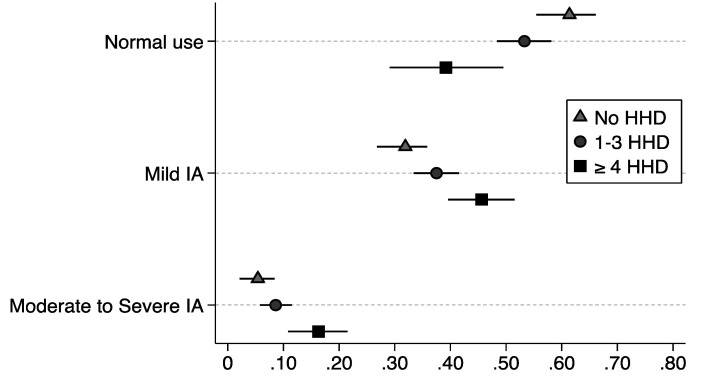
Predicted probability with 95% confidence intervals of normal Internet use, mild Internet addiction, and moderate to severe Internet addiction for ACE groups, after adjusting for all covariates (i.e., age, gender, ethnicity, SES, COVID-19 employment loss, depression).

The results of the main-effects models are presented in Table 3. Students who reported 1–3 household stressors had higher proportional odds of mild Internet addiction vs. normal or moderate to severe than students who did not report any household dysfunction (AOR: 1.39, 95% CI: 1.05–1.83), whereas students who reported ≥4 household stressors had over twice the proportional odds of having moderate to severe Internet addiction vs. mild or normal Internet use (AOR: 2.46, 95% CI: 1.52–3.96), adjusting for covariates. In regard to peer social support, for every additional unit increase in the peer support score, there was a decrement in the proportional odds of mild or moderate to severe IA vs. normal Internet use (AOR: 0.91, 95% CI: 0.86–0.97). As seen in Figure 1, the probability of normal Internet use is far higher among students who report no HHD and relatively low among students with ≥4 household stressors, whereas the likelihood of mild and moderate to severe Internet use increases incrementally among students with 1–3 household stressors and ≥4 household stressors compared to students with no history of HHD.

There was a similar pattern of results in models assessing the association between HHD and PSU. Students who reported 1–3 household stressors had higher odds of PSU (AOR: 1.40, 95% CI: 1.02–1.93) than their peers who did not report HHD, and students who had ≥4 household stressors had approximately twice the odds (AOR: 2.03, 95% CI: 1.21–3.40) of PSU than their peers. There was no association between peer support and PSU in the main-effects models. 

**Figure 2:**
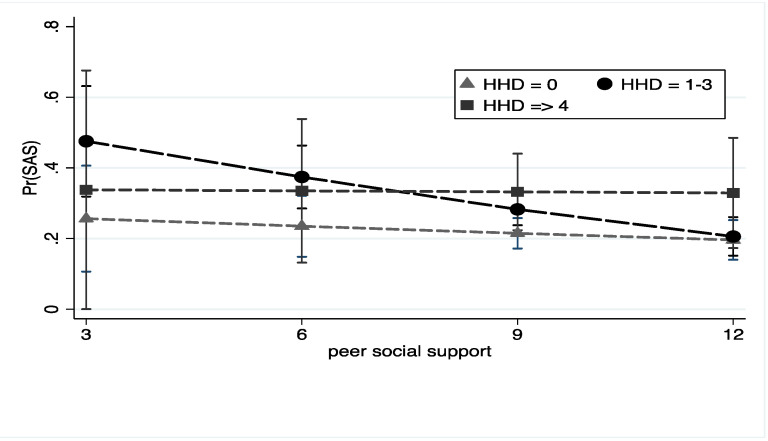
HHD, peer social support, and problematic smartphone use. Note: Figure depicts the predicted probability with 95% confidence intervals of problematic smartphone use among students with a history of HHD at low and high levels of social support. All models adjusted for age, gender, ethnicity, SES, COVID-19 employment loss, and depression.

We also tested hypotheses that perceived that social support from friends would attenuate the negative effects of HHD for PIU and PSU. Social support from friends did not moderate the association between HHD and any level of PIU. However, as seen in Figure 2, higher levels of perceived peer social support offset the negative effect of HHD for PSU among students who reported 1–3 household stressors, but not among those reporting ≥4, adjusting for all covariates.

## 4. Discussion

This is one of the first studies to examine the association between cumulative HHD and problematic Internet and smartphone use in a diverse, American college sample. Over 50% of the sample reported at least one household stressor, and nearly 10% acknowledged four or more, estimates that are similar to those reported by national and community surveillance efforts [98,99]. Approximately 25% of students reported problematic smartphone use, 35% of students scored in the mild, and 10% scored in the moderate to severe PIU category, respectively. These estimates are also within range of those reported among young people and college students [100,101,102,103], although most studies have originated outside of the US.

Our primary hypotheses that students with a history of household dysfunction, and especially students with multiple co-occurring household stressors, would be at greater risk for problematic Internet and smartphone use were supported. This is concerning given that the SAS-SV and IAT assess the presence of life disturbance, difficulty concentrating, and an inability to meet obligations [14,104]—symptoms characteristic of addiction disorders [41,43]. The digital era and widespread use of the Internet and smartphones pose a risk of misuse for all young people, but especially individuals who are vulnerable to maladaptive coping and addictive behaviors. Scholars have argued that Internet and digital devices offer distractions and online activities that may be particularly attractive for young people seeking temporary relief from negative affect or feelings of distress [105,106]. This may be especially true for young adults who contend with the psychological and emotional impact of household dysfunction (i.e., parental mental illness or substance use) while they assume greater responsibility, manage academic obligations, and plan for the future. The joint effects of these developmental challenges and traumatic stressors can strain a young person’s available resources [107,108] and increase the likelihood of using digital devices and engaging in online activities to reduce stress and negative emotions [104,109,110]. However, compulsive digital device and Internet use as a means to relieve symptoms of psychological distress can actually exacerbate negative affect [109,111] and ensnare a young person in a cycle of increasing Internet and smartphone use to manage mood without ever addressing, or resolving, the underlying causes. Moreover, the wide availability and need for Internet and digital devices in daily life present challenges for the treatment and prevention of PIU and PSU [6].

The graded relationship between levels of HHD and PSU and PIU among this population is consistent with research demonstrating that early life adversity increases vulnerability for maladaptive coping behaviors, theorized to be a result of the ACE-related dysregulation of reward pathways and deficits in emotional and cognitive processing [112,113] that become more pronounced as the number of adversities increase [46]. In the context of PIU and PSU, there is burgeoning evidence that in comparison to individuals who do not report PSU or PIU, individuals who score as compulsive users on diagnostic instruments have significant structural and neural functioning differences that are similar to those of persons with substance use disorders [114,115]. Our results are preliminary, yet they align with and expand evidence that ACE are a shared risk factor for many of the behavioral addictions that compromise health, educational attainment, and life course wellbeing [98]. Although the mechanism through which any individual ACE affects health may be unique, the impact of a constellation of household stressors on physiological and psychological processes likely represents a common pathway to a range of long-term behavioral health issues [46]. If left unaddressed, a history of cumulative HHD can jeopardize academic performance and the degree to which students can leverage new academic and professional opportunities [45,59,116,117,118]—critical components of protecting the next generation from the harmful health effects of ACE.

In partial support of our hypothesis that perceived social support from peers would offset the negative effects of HHD, our results suggest that perceived peer social support can have promotive and protective effects for PIU and PSU, respectively. Perceived peer support was associated with lower odds of PIU, and although we did not assess online vs. offline support, this mirrors work that suggests strong interpersonal ties and perceived support from friends reduce risk for PIU [9,119,120]. High levels of perceived peer support, although not statistically associated with PSU in the main-effects model, offset the negative effects of HHD for PSU among students experiencing between one and three household stressors. This finding aligns with research demonstrating that social support can limit the physiological arousal and stress reactivity associated with traumatic circumstances and promote resilient functioning [121,122]. Despite the protective effects of strong bonds with teachers, peers, and members of important community groups for ACE-exposed populations [85,123,124], support from peers may be insufficient or need to be paired with direct services or additional individual or social assets to mitigate the negative effects of high levels (≥4 HHD) of adversity for PSU and PIU.

Given the importance of the college years for later-life advantage and the substantial evidence that ACE can jeopardize young adult mental and behavioral health, addressing the pervasive, long-term, negative consequence of these traumatic stressors for positive adjustment is imperative. College communities serve a diverse cross-section of young adults and are an opportune setting to provide support services for vulnerable students as they transition from adolescence to adulthood. The benefits of trauma-informed care for other segments of the population [125,126,127] make a persuasive argument for prioritizing prevention and intervention initiatives in settings that foster young peoples’ development [128,129] and promote positive health outcomes. An important area of future research is to identify individual, peer, community, and cultural assets that can offset the negative effects of HHD specifically, or ACE generally, for PSU, PIU, and other behavioral addictions and how these can be leveraged in prevention programs tailored to young adults.

### Limitations

The present study had several limitations. First, these data are cross-sectional, do not support causal conclusions, and are generalizable only to college students attending universities in diverse, urban settings similar to that of Southern California. Second, although demographic profiles resembled college profiles, it is likely that the highest-risk students did not participate in the survey, and therefore, we underestimated the prevalence of PIU, PSU, HHD, and the association between HHD and PIU and PSU. Third, due to the limited number of students reporting ≥4 HHD, we may have underestimated the potential protective effects of peer social support for PSU. Fourth, we assessed household dysfunction retrospectively; however, since survey items explicitly referred to events prior to age 18, whereas PIU and PSU assessed recent behaviors, reverse temporality is unlikely. Fifth, the SAS-SV does not include items assessing mood or relapse, and although the threshold for PSU recommended by SAS-SV developers is widely used, it does not represent an established diagnostic criterion. Sixth, we did not ask whether students’ peer support sources were online or offline and thus were not able to determine whether online activities enhance students’ perceptions of having meaningful and supportive social ties. Lastly, our sample was predominantly female and may have led to over or underestimating the strength of associations.

Despite these limitations, this study is among the first to examine the relationship between HHD and PIU and PSU among a diverse sample of American college students. Further research among college populations using longitudinal designs to replicate these findings is suggested.

## 5. Conclusions

Given the high prevalence of ACE, PIU, and PSU in college populations, and based on the extraordinary net benefits of a college education, which may actually mitigate the effects of early life adversity [130], funding prevention and support services in higher education could substantially reduce the trauma-related mental and behavioral public health burden. Campus communities could play a pivotal role in promoting resilience during the transition from adolescence to adulthood by raising awareness of the risks and consequences of PIU and PSU, by encouraging students to access support services that can help them to cope with familial adversity, and by promoting the development of meaningful social bonds.

## Figures and Tables

**Table 1:** Summary of missing data.

*N* = 1273	Completed	Imputed	Total
Sex	1027	0	1027
Age	1027	0	1027
Ethnicity	973	54	1027
COVID work loss	1027	0	1027
Depression	1027	0	1027
Peer Support	991	36	1027
ACE: Household Dysfunction	1027	0	1027
Problematic Internet Use (PIU/IA)	981	46	1027
Problematic Smartphone Use (PSU/SA)	950	77	1027

Note: Complete + Imputed = Total; imputed is the minimum across m of the number of filled-in observations.

**Table 2:** Sample characteristics (*N* = 1027).

Biological Sex	Frequency (%) or M (SD)
Female	804 (78.32)
Male	223 (21.68)
**Age**	
17–21	417 (40.57)
22–29	475 (46.24)
30+	135 (13.20)
**Ethnicity**	
Mexican	392 (38.18)
Other and Bi-/Multiracial	172 (16.74)
Non-Hispanic White	169 (16.48)
Asian	125 (12.20)
Other Hispanic	124 (12.03)
Non-Hispanic Black	45 (4.37)
**Perceived Peer Social Support (MSPSS)**	9.59 (2.24)
**COVID-19 Work Loss (Hours)**	5.09 (13.49)
**Depressive Symptoms (CES_D)**	
Low risk of depression	631 (61.46)
Significant depressive symptoms	396 (38.54)
**Household dysfunction (ACE)**	
No ACE	450 (43.82)
1–3 ACE	484 (47.13)
4+ ACE	93 (9.06)
**Problematic Internet Use (PIU)**	
Normal use	573 (55.80)
Mild dependence	352 (34.32)
Moderate or severe dependence	102 (9.88)
**Problematic Smartphone Use (PSU)**	
Low risk of PSU	777 (75.68)
High risk of PSU	250 (24.32)

**Table 3:** Association between household dysfunction (HHD) and problematic Internet and smartphone use (*N* = 1027).

	Internet Addiction (IA) AOR (95% CI)	Problematic Smartphone Use (PSU) AOR (95% CI)
**Household Dysfunction (HHD)**		
1–3 ACE	1.39 (1.05–1.83) *	1.40 (1.02–1.93) *
≥4 ACE	2.46 (1.52–3.96) **	2.03 (1.21–3.40) **
**Age**	0.93 (0.90–0.96) **	0.96 (0.93–0.99) *
**Ethnicity**		
Non-Hispanic Black	0.94 (0.47–1.89)	1.11 (0.51–2.42)
Mexican	0.51 (0.35–0.75) **	0.64 (0.40–1.00)
Other Hispanic	0.73 (0.44–1.20)	0.62 (0.34–1.12)
Asian	1.54 (0.95–2.47)	1.28 (0.72–2.28)
Other and Bi-/Multiracial	0.89 (0.58–1.38)	1.02 (0.63–1.66)
**Biological Sex**		
Female	1.09 (0.78–1.52)	1.06 (0.73–1.55)
**Perceived Social Support**	0.91 (0.86–0.97) **	0.93 (0.87–1.00)
**COVID-19 Work Loss**	0.96 (0.99–1.01)	0.99 (0.98–1.00)
**Depression (CES_D)**	1.00 (0.99–1.01)	1.00 (0.99–1.01)

**Note** AOR: Adjusted Odds Ratio. Reference groups for ACE: No ACE; Ethnicity: Non-Hispanic White; Biological sex: Male. * = *p* < 0.05, ** *p* = < 0.01.
